# Anogenital Distance from Birth to 2 Years: a Population Study

**DOI:** 10.1289/ehp.0900881

**Published:** 2009-07-13

**Authors:** Ajay Thankamony, Ken K. Ong, David B. Dunger, Carlo L. Acerini, Ieuan A. Hughes

**Affiliations:** 1 Department of Paediatrics, University of Cambridge, Cambridge, United Kingdom; 2 MRC Epidemiology Unit, Institute of Metabolic Science, Cambridge, United Kingdom

**Keywords:** anogenital distance, cryptorchidism, endocrine-disrupting chemicals, endocrine disruption, hypospadias

## Abstract

**Background:**

Anogenital distance (AGD) is sexually dimorphic in rodents and humans, being 2- to 2.5-fold greater in males. It is a reliable marker of androgen and antiandrogen effects in rodent reproductive toxicologic studies. Data on AGD in humans are sparse, with no longitudinal data collected during infancy.

**Objective:**

This study was designed to determine AGD from birth to 2 years in males and females and relate this to other anthropometric measures.

**Materials and Methods:**

Infants were recruited from the Cambridge Baby Growth Study. AGD was measured from the center of the anus to the base of the scrotum in males and to the posterior fourchette in females. Measurements were performed at birth and at 3, 12, 18, and 24 months of age.

**Results:**

Data included 2,168 longitudinal AGD measurements from 463 male and 426 female full-term infants (median = 2 measurements per infant). Mean AGD (± SD) at birth was 19.8 ± 6.1 mm in males and 9.1 ± 2.8 mm in females (*p* < 0.0001). AGD increased up to 12 months in both sexes and in a sex-dimorphic pattern. AGD was positively correlated with penile length at birth (*r* = 0.18, *p* = 0.003) and the increase in AGD from birth to 3 months was correlated with penile growth (*r*= 0.20, *p* = 0.001).

**Conclusion:**

We report novel, longitudinal data for AGD during infancy in a large U.K. birth cohort. AGD was sex dimorphic at all ages studied. The availability of normative data provides a means of utilizing this biological marker of androgen action in population studies of the effects of environmental chemicals on genital development.

The trends in male reproductive health characterized by increases in the incidence of cryptorchidism, hypospadias, testicular tumors, and declining semen quality observed in many countries have been the subject of a number of recent reviews (Acerini and Hughes 2006; Diamanti-Kandarakis et al. 2009; Foresta et al. 2008; Virtanen et al. 2005). Geographic differences in these trends and prevalence rates point to the causative role of environmental factors (Toppari et al. 1996). These disorders are shown to be risk factors for each other, with a common link being an abnormality of fetal testis development resulting in a testicular dysgenesis syndrome (Wohlfahrt-Veje et al. 2009). The environmental factors are postulated to be compounds that act as endocrine disruptors; they affect the developing testes and lead to male reproductive tract anomalies by altering the balance of androgen and estrogen action. Indeed, the American Endocrine Society has highlighted endocrine-disrupting chemicals (EDCs) as a priority area for increased research funding when submitting evidence to the new U.S. president’s administration in 2009 (Diamanti-Kandarakis et al. 2009). Although evidence for the effects of EDCs can be readily ascertained in wildlife and experimental animals, a challenge remains in defining a sensitive marker in humans that reflects exposure to EDCs such as phthalates (Vandenberg et al. 2009).

A sensitive marker used by reproductive toxicologists in rodent experiments is anogenital distance (AGD). It is measured from the center of anus to the genital tubercle and is sexually dimorphic, being 2- to 2.5-fold longer in males. Numerous studies attest to its validity as a sensitive marker for the effects of *in utero* exposure to androgens and chemicals with antiandrogen effects (McIntyre et al. 2001; Mylchreest et al. 2000; Wolf et al. 2004). Consequently, AGD has been identified as one of the end points in U.S. Environmental Protection Agency guidelines for reproductive toxicity studies in humans (Arbuckle et al. 2008). Nevertheless, population studies of AGD in humans are limited to measurement at birth (Romano-Riquer et al. 2007; Salazar-Martinez et al. 2004). It has been proposed that dynamic changes in the AGD may be particularly sensitive to the effects of exposure to EDCs (Arbuckle et al. 2008). The aims of the present study were to generate normative data of AGD in infants from birth to 2 years and to determine its association with other anthropometric measurements.

## Materials and Methods

### Population

Infants were participants of the Cambridge Baby Growth Study, an ongoing prospective, longitudinal study established in 2001 to characterize hormonal, genetic, and environmental influences on infant growth and early male reproductive development. Measurement of AGD was included in the study protocol from 2006. Mothers were recruited from the maternity unit of Addenbrooke’s Hospital, Cambridge (UK), at around 12 weeks of gestation when attending for their routine antenatal ultrasound scan. The unit conducts about 5,000 deliveries per year and supports a mixed urban and rural community in South Cambridgeshire. Information about maternal lifestyle was collected using self-administered questionnaires. Detailed analysis of this information is the subject of a separate study. The research protocol was approved by the Cambridge Local Research Ethics Committee, and the study was conducted in accordance with the International Conference on Harmonization standards for Good Clinical Practice. The mothers gave written informed consent for themselves and their infants to participate in the study before the recruitment. The infants were measured at birth either at the hospital or at home and subsequently during infancy in a dedicated follow-up research clinic by a team of four research nurses.

### Measurements

Infants were measured at birth and at 3, 12, 18, and 24 months of age. Measurements of AGD, weight, length, and penile length were collected, and the external genitalia were examined for any abnormalities.

**Figure 1:**
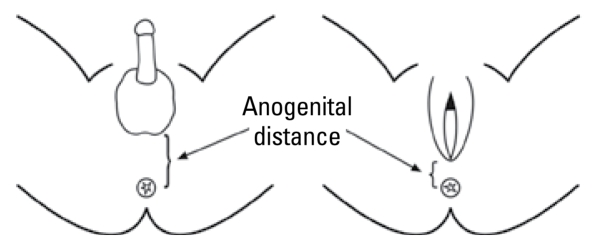
Schematic diagram of anatomical landmarks to measure AGD. (Reproduced from Salazar-Martinez et al. 2004, originally published in *Environmental Health* by Biomed Central).

A modified version of a previously published method by Salazar-Martinez et al. (2004) was used to measure AGD. The measurement was taken from the center of anus to the junction of smooth perineal skin and rugated skin of the scrotum in males, and to the posterior convergence of the fourchette in females (Figure 1) using Vernier calipers (DialMax; Wiha Premium Tools, Schonach, Germany) that read in increments of 0.1 mm. The infant was placed on the dorsal decubitus position with both hips flexed and light pressure exerted on the thighs. The calipers were held in the right hand, with the handle pointing up, and tilted slightly toward the infant’s head so that the measuring surfaces were adjacent to the landmarks. Care was taken to avoid stretching of the perineum if the scrotum needed to be lifted to identify the perineoscrotal junction. Penile length was measured from the lower edge of the pubic bone to the tip of the flaccid penis using Vernier calipers. Three consecutive measurements were taken at each assessment, and the average was used for analysis. The calipers were calibrated three times a year.

The research nurses received regular training within the department and participated in annual quality control exercises when an anthropometrist supervised their measurement techniques. The interobserver variability was assessed by repeated measurements in 14 infants, and the technical error of measurement (TEM) was calculated (Ulijaszek and Kerr 1999). The absolute and relative TEM values were 3 mm and 9.6% in males and 1 mm and 5.7% in females, respectively.

### Data analysis

The gestational age was calculated from the last menstrual period. Preterm (gestation < 37 weeks) and low-birth-weight (birth weight < 2,500 g) infants were excluded from analysis. Subjects whose measurements were performed beyond a 4-week interval of the proposed date of follow-up were also excluded. Differences between variables were determined by using analysis of variance (version 15.0; SPSS for Windows, Chicago, IL, USA). We used Pearson’s correlation coefficient to estimate the strength of associations. We assessed the longitudinal change in AGD by calculating the difference between neighboring time points in infants who were measured on both occasions. AGD and other variables were assessed by multiple linear regression models using weight, length, penile length, gestational age, and age at examination as independent variables. *p*-Values of < 0.05 were considered statistically significant. Centile curves were created using lmsChart-Maker Light version 2.3 (T. Cole, H. Pan, Medical Research Council, London, UK). Results are presented as mean (± SD) except as specified.

## Results

We assessed AGD in 889 infants (463 male, 426 female). Twenty infants were born of twin gestation. Birth weight of infants were similar to 1990 British reference data, in the form of mean standard deviation score [SDS (± SD)]: male, 0.08 ± 0.88; female, 0.14 ± 0.88 (Freeman et al. 1995). However, birth length was higher in both males (0.32 ± 1.55) and females (0.4 ± 1.1) than in the reference data. Mean gestational ages for males and females were 40.1 ± 1.2 and 40.0 ± 1.2 weeks, respectively. Table 1 lists the main characteristics of the mothers in this study. The follow-up rates in Cambridge Baby Growth Study expressed as the percentage of expected attendance as calculated from the preceding visit to the actual attendance at each visit were 80% at 3 months, 85.8% at 12 months, 90.3% at 18 months, and 94.7% at 24 months of age.

### AGD measurements

**Figure 2:**
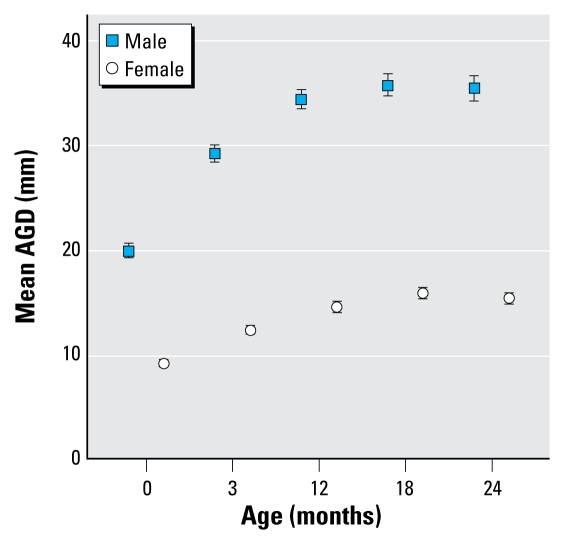
Mean ± 95% CI AGD measurements in males and females from birth to 2 years of age.

**Figure 3:**
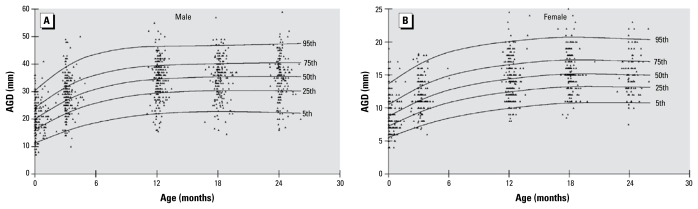
Centile curves and data plots for AGD in males (*A*) and females (*B*) up to 2 years of age. Data points represent individual measurements.

Measurements of AGD were obtained from 2,168 visits in 889 infants [median = 2 (range, 1–5) measurements per infant] (Table 2). Males had a greater AGD than did females at birth and at all other time points (*p* < 0.0001) (Figure 2). At birth, mean AGD in males was approximately 2-fold higher than in females, and this difference persisted to the same extent at all stages of the study. In both sexes, AGD increased rapidly in the first 12 months of life and then gradually plateaued. Figure 3 shows this trend as the distribution of the measurements superimposed on smoothed centile lines. Analysis of longitudinal changes in AGD showed that no further rise in AGD after 18 months of age (Table 3). AGD at birth was associated with subsequent AGD measurements at ages 3, 12, and 24 months in males, but only at 3 months of age in females (Table 4).

### Association of AGD with body size

At each time point, we analyzed AGD for correlation with respective measures of body size. In males, AGD was correlated with weight at 3 months and with length at 3 and 12 months of age (Table 5). In a linear regression model using weight, length, age, gestational age, and penile length as predictors for AGD, we observed a positive correlation only with weight at birth (*r* = 0.16, *p* = 0.006). In females, AGD was correlated with weight at birth, 3, 12, and 18 months and with length at 18 and 24 months of age. When adjusted for other variables in a linear regression model using weight, length, gestational age, and age as independent variables the positive association persisted only for length at 24 months of age (*r* = 0.21, *p* = 0.014).

### Correlation with penile length

AGD was correlated with penile length only at birth (*r* = 0.18, *p* = 0.003). We also observed a correlation between the increase in AGD between birth and 3 months of age and an increase in penile length during the same period (*r* = 0.20, *p* = 0.001). Neither of these parameters showed any correlation with the increase in weight or length during the same period. In a linear regression model using weight, length, gestational age, penile length, and age as variables, birth weight and penile length were the only predictors of AGD at birth [β = 0.31, 95% confidence interval (CI), 0.09–0.52, *p* = 0.006; and β = 0.25, 95% CI, 0.09–0.42, *p* = 0.003, respectively]. Penile growth was the only predictor for the increase in AGD during the first 3 months of life (β = 0.28; 95% CI, 0.09–0. 47, *p* = 0.004) in a linear regression model using increase in weight and length as independent variables. Similarly, in females increases in weight and length from birth to 3 months of age were not correlated with an increase in AGD during the same period.

Fourteen male infants had genital abnormalities at birth (incomplete testicular descent, 13; hypospadias, 2). In a preliminary analysis, the mean AGD in 13 infants with incomplete testicular descent at birth was shorter than in males with normal testis descent (17.5 mm vs. 19.9 mm) (Table 6). However, this difference did not reach statistical significance. Seven of these infants had one or both testes in a high scrotal position, in two the testes were suprascrotal in position, and four infants had nonpalpable testes. We also noted a similar trend in AGD measurements in infants with incomplete testicular descent at 3, 12, and 24 months of age. Two infants were born with hypospadias; their mean AGD was also shorter than in normal males (15.5 mm vs. 19.8 mm).

## Discussion

This study confirms sexual dimorphism of AGD at birth that was maintained to the same degree during the first 2 years of life. In males, AGD was related to penile length and penile growth and hence may be useful as a marker of androgen action.

Perineal growth and caudal migration of the genital tubercle in male rodents are androgen (dihydrotestosterone)-dependent processes (Bowman et al. 2003). Consequently, measurement of AGD is considered to be a sensitive index of prenatal androgen action and hence could be used in humans as a marker of the antiandrogen effects of EDCs. The external genitalia in the human are clearly sexually dimorphic at birth, with the genital tubercle differentiated into a penis or clitoris. In previous human studies, different protocols were used to measure AGD. The first population study in both sexes measured AGD from the center of anus to the base of the scrotum in males and to the posterior fourchette in females (Salazar-Martinez et al. 2004). Investigators have also measured AGD in males from the anus to the anterior and posterior base of the penis, akin to the measurement used in rodents (Hsieh et al. 2008; Romano-Riquer et al. 2007). We adopted the former protocol in the present study because the anatomical landmarks are easily identifiable. The anterior anatomical landmark of the measurement in our study indicates the location of the caudal border of the genital swelling, the anlagen that differentiates as the labia majora in females and the scrotum in males.

The mean AGD in males at birth in the study (19.8 mm) is similar to the measurements published by Romano-Riquer et al. (2007; 19.1 mm) and Salazar-Martinez et al. (2004; 21 mm) in a Mexican population. In females, the mean AGD at birth (9.1 mm) was similar to that reported by Salazar-Martinez et al. (2004; 11 mm) in Mexico and Callegari et al. (1987; 10.9 mm) in California, but much less than the mean value (16.1 mm) reported in Israel (Phillip et al. 1996). Although ethnic differences have yet to be reported, systematic errors in measurement may also explain the variation. Our results are similar to that reported in the two Mexican studies despite the greater mean birth size of the U.K cohort (males, 3.59 kg; females, 3.47 kg) compared with the Mexican study populations (males, 3.28 kg; and males, 3.06 kg; females, 3.07 kg).

AGD rapidly increased from birth to 12 months of age and then gradually plateaued, resulting in a 70% increase in this measurement during the first 2 years of life in both sexes. The reported length of the perineum measured from the anterior anal margin to the posterior fourchette in adult females (31.3 mm ± 8.5) is almost double the mean AGD at 2 years of age (Lloyd et al. 2005). Even though the timing of this change remains speculative, the pattern of AGD increase during infancy suggests that a significant component of the remaining perineal growth is likely to occur at puberty along with growth of the external genitalia. Comparable normative data in adult males have not yet been reported. The correlation of AGD at birth with subsequent measurements during infancy was modest and reduced with increasing age. Technical difficulties with measurements in older infants (Swan et al. 2005), interobserver variability, and the relatively small number of infants available for analysis at later ages may explain this observation.

In rodents, birth weight is not a strong predictor of AGD (Gallavan et al. 1999). In the present study, any association with body weight in males disappeared after 3 months of age. The correlation with birth weight was weaker in our study compared with that found in a Mexican population. Socioeconomic factors and smaller maternal size resulting in relative growth restriction of the fetus may explain the difference. Because body weight is an inconsistent predictor of AGD during infancy, we suggest that adjusting AGD for body size may render the anthropometric measurements less reliable. Correlation between AGD and weight and length are less consistent during the first 2 years of life. The inconsistency may be related to the variable sample size at each time point, and further studies are required to confirm these observations.

The degree of sexual dimorphism in AGD was consistent at all stages of the 2-year longitudinal study. The association of AGD with the penile length at birth and penile growth in the first 3 months may reflect the effect of prenatal androgens and the postnatal surge in testosterone secretion, respectively. The reported strong association of penile growth in early infancy with serum testosterone levels at 3 months of age (Boas et al. 2006) provides indirect evidence of a postnatal change in AGD in response to androgens. A shorter AGD has been found in infants with undescended testes (Swan et al. 2005) and hypospadias (Hsieh et al. 2008) based on cross-sectional studies. We observed similar trends in AGD in such infants in our study who had these common male birth defects.

Any evidence in humans of changes in AGD resulting from prenatal exposure to androgens and EDCs is limited. Labioscrotal fusion and increased anogenital ratio (ratio of AGD to anoclitoral distance) was reported in three female infants with virilizing congenital adrenal hyperplasia (Callegari et al. 1987). A decreased AGD was reported in male infants whose mothers had been exposed prenatally to EDCs based on high levels of phthalates in maternal urine (Swan et al. 2005); in this cross-sectional study in males 2–36 months of age, AGD was measured from the anus to the anterior base of the penis and was corrected for weight by calculating an anogenital index (AGI = AGD/weight). Furthermore, the AGI was shorter in males with incompletely descended testes, smaller penile size, and altered scrotal development. A reduced anal position index, defined as the ratio of AGD to the distance between the coccyx and the scrotum, was found in male infants whose mothers had high first-trimester blood levels of 1,1-dichloro-2,2-bis(*p*-chlorophenyl)ethylene (DDE), a metabolite of the pesticide 1,1,1-trichloro-2,2-bis(*p*-chlorophenyl)ethane (DDT) (Torres-Sanchez et al. 2008). In contrast, a large population study of infants at birth did not demonstrate a similar relationship with maternal blood DDE levels (Longnecker et al. 2007).

Measurement of AGD offers a potentially more sensitive marker of the effects of prenatal exposure to EDCs in humans compared with possible indices of endocrine-disrupting effects such as cryptorchidism and hypospadias. The longitudinal normative data from this study is fundamental to applying the technique of AGD measurement for future cross-sectional population studies of the effects of EDCs in human populations.

## Conclusions

The present population-based prospective study reports for the first time normative data for AGD in both sexes from birth to 2 years of age. We observed a sex-dimorphic pattern at all ages studied. Growth of the perineum as determined by measurement of the AGD was characterized by a rapid increase in the first 12 months of life, followed by gradual plateauing in both sexes. The association with penile length indicates that AGD may be influenced by both prenatal and early postnatal androgen secretion. By the same token, any antiandrogen effects during this period of human development may be reflected in decrements in AGD measurements. The availability of longitudinal normative data for AGD now provides a means to determine the reliability of this marker as a sensitive indicator of the effects of EDCs in human populations.

## Figures and Tables

**Table 1:** Characteristics of mothers participating in the study.

Characteristic	Results
Age (years)	33.3 (SD, 4.2)
Prepregnancy weight (kg)	66.6 (SD, 13.3; SDS, 0.18)
Height (cm)	166 (SD, 7.1; SDS, 0.46)
Parity (%)	
1	43.7
2	42.7
> 2	13.6
Caucasian ethnicity (%)	97.2
Smoking (%)	2.2
Alcohol consumption (%)	34
Strong alcohol and spirits (%)	1.8

**Table 2:** Characteristics of infants included in the study (mean ± SD).

Characteristic	Birth	3 months	12 months	18 months	24 months
Male					
No.	285	259	241	187	169
Age (months)	0.1 ± 0.2	3.2 ± 0.3	12.2 ± 0.3	18.1 ± 0.3	24.1 ± 0.3
Weight (kg)	3.59 ± 0.46	6.39 ± 0.76	10.27 ± 1.06	11.66 ± 1.20	12.92 ± 1.35
Length (cm)	51.6 ± 2.0	62.0 ± 2.4	76.7 ± 2.5	83.0 ± 2.8	88.4 ± 3.3
Penile length (cm)	3.04 ± 0.43	3.42 ± 0.52	3.69 ± 0.58	3.96 ± 0.69	4.09 ± 0.65
AGD (mm)	19.8 ± 6.1	29.0 ± 6.8	34.2 ± 7.5	35.5 ± 7.3	35.2 ± 7.8

Female					
No.	279	220	223	168	137
Age (months)	0.1 ± 0.2	3.2 ± 0.3	12.2 ± 0.3	18.1 ± 0.3	24.1 ± 0.3
Weight (kg)	3.47 ± 0.43	5.88 ± 0.66	9.56 ± 1.08	11.08 ± 1.26	12.27 ± 1.68
Length (cm)	50.8 ± 2.0	60.5 ± 2.2	75.0 ± 2.5	81.5 ± 3.2	86.8 ± 3.0
AGD (mm)	9.1 ± 2.8	12.2 ± 2.9	14.5 ± 3.3	15.8 ± 3.5	15.3 ± 3.0

**Table 3:** Increase in AGD (95% CIs) between neighboring ages of measurement (mm).

Measure	0–3 months	3–12 months	12–18 months	18–24 months
Male				
No.	204	165	156	125
Mean	8.45 (7.37 to 9.52)	4.79 (3.53 to 6.05)	0.95 (−0.39 to 2.28)	−0.25 (−1.96 to 1.46)

Female				
No.	191	131	146	107
Mean	3.28 (2.8 to 3.77)	1.29 (0.63 to 1.95)	1.08 (0.4 to 1.77)	−0.60 (−1.40 to 0.21)

Values are mean increase in AGD (95% CI).

**Table 4:** Correlation between AGD at birth with subsequent measurements.

Measure	3 months	12 months	18 months	24 months
Male				
No.	204	131	82	52
*r* (*p-*value)	0.30* (< 0.0001)	0.24 *(0.003)	0.15 (0.08)	0.26* (0.03)

Female				
No.	191	130	85	49
*r* (*p-*value)	0.26* (< 0.0001)	0.07 (0.21)	0.14 (0.10)	0.07 (0.31)

* *p* < 0.05.

**Table 5:** Correlation of AGD with other anthropometric measures.

Measure	Birth	3 months	12 months	18 months	24 months
Male					
Weight	0.10 (0.07)	0.16* (0.01)	−0.13 (0.84)	0.09 (0.24)	0.15 (0.06)
Length	0.01 (0.83)	0.14* (0.03)	0.02 (0.77)	0.10 (0.17)	0.18* (0.02)
Penile length	0.18* (0.003)	0.11 (0.09)	0.07 (0.30)	0.10 (0.16)	0.15 (0.05)

Female					
Weight	0.14* (0.02)	0.15* (0.03)	0.14* (0.04)	0.16* (0.04)	0.17 (0.05)
Length	0.08 (0.22)	0.02 (0.73)	0.09 (0.17)	0.17* (0.03)	0.30* (0.001)

Values are correlation coefficient (*r*) (*p-*values).

* *p* < 0.05.

**Table 6:** AGD (mm) in males with cryptorchidism and hypospadias (mean ± SD).

Category	Birth	3 months	12 months	18 months	24 months
Normal males	19.8 ± 6.1	29.0 ± 6.8	34.2 ± 7.5	35.5 ± 7.3	35.2 ± 7.8
Cryptorchidism	17.5 ± 6.9	27 ± 7.3	33.5 ± 6.7	—	31.0 ± 4.6
	(*n* = 13)	(*n* = 5)	(*n* = 6)	—	(*n* = 3)
Hypospadias	15.5 ± 2.1	—	30 ± 11.5	—	—
	(*n* = 2)	—	(*n* = 4)	—	—

—, no data.
